# CircCSPP1 Functions as a ceRNA to Promote Colorectal Carcinoma Cell EMT and Liver Metastasis by Upregulating COL1A1

**DOI:** 10.3389/fonc.2020.00850

**Published:** 2020-06-16

**Authors:** Qingyuan Wang, Linsen Shi, Kui Shi, Bo Yuan, Gang Cao, Chenchen Kong, Jun Fu, Zhongsong Man, Xu Li, Xuanfeng Zhang, Yifei Feng, Xinchun Jiang, Xinhui Zhang, Jun Yan, Xinyong Wu, Yueming Sun

**Affiliations:** ^1^Center of Hepatobiliary Pancreatic Disease, Xuzhou Central Hospital, Xuzhou, China; ^2^Department of General Surgery, The First Affiliated Hospital of Nanjing Medical University, Nanjing, China; ^3^Department of Gastrointestinal Surgery, The Affiliated Hospital of Xuzhou Medical University, Xuzhou, China; ^4^Center of Hepatobiliary Pancreatic Disease, Beijing Tsinghua Changgung Hospital, Beijing, China; ^5^Department of General Surgery, Xuzhou Central Hospital, Xuzhou, China

**Keywords:** circRNA, CeRNA, EMT, COL1A1, metastasis

## Abstract

The aberrant regulation of circular RNAs (circRNAs), ring structures formed by exon or intron backsplicing, has been identified as a novel characteristic of multiple cancers. However, the role of circRNAs in colorectal carcinoma remains to be elucidated. In the present study, we investigated the mRNA level and the promoting effect of circRNA CSPP1 (circCSPP1) in colorectal carcinoma liver metastasis. By bioinformatic analysis of 10 paired samples of colorectal carcinoma and adjacent mucosal tissues, we identified circCSPP1 as a significantly upregulated circRNA in colorectal carcinoma tissues, and its upregulation was correlated with a higher M stage. The gain- and loss-of-function assays revealed that circCSPP1 promotes the migration and invasion of colorectal carcinoma cells *in vitro* and *in vivo*. Mechanistically, similar miRNA response elements are shared between circCSPP1 and COL1A1. We demonstrated that circCSPP1 upregulates the mRNA levels of COL1A1, which plays a pivotal role in the process of epithelial–mesenchymal transition (EMT), by competitively binding to miR-193a-5p and preventing miR-193a-5p from decreasing the expression of COL1A1. In conclusion, this finding indicates that circCSPP1 may act as a promising therapeutic target by regulating the EMT process in colorectal carcinoma via activation of the circCSPP1/miR-193a-5p/COL1A1 axis.

## Introduction

Colorectal carcinoma (CRC) is the third most common cancer, with 1.8 million newly diagnosed cases and 0.88 million deaths annually (1, 2). Exploring the underlying mechanisms of CRC growth and metastasis is crucial to developing more effective treatment options. Accumulating evidence indicates that CRC results from multiple complex factors that include genetic, molecular, and epigenetic alterations (3, 4).

Circular RNAs (circRNAs) are defined as a class of non-protein-coding transcripts and are characterized by closed-loop structures whose potential regulatory roles have attracted enormous interest in cancer research recently (5). The mechanisms by which circRNAs function as competing endogenous RNAs (ceRNAs) have been the most studied in circRNA-based cancer research. Among these, diverse cellular RNA mechanisms, including protein interactions (6), organization of nuclear architecture (7), regulation of post-translational modifications (PTMs) (8), and protein translation (9), have also been the subject of circRNA investigations. For example, circNSUN2, which can form an RNA–protein ternary complex with IGF2BP2 and HMGA2, promotes CRC liver metastasis by enhancing the RNA stability of HMGA2 (10).

COL1A1, a member of the collagen family, was reported to be involved in the carcinogenesis of several tumor types in recent years (11–18). Along with COL1A2, COL1A1 is composed of two chains of type I collagen (19, 20). The dysregulation of COL1A1 and COL1A2 is thought to be associated with changes in the extracellular matrix (ECM) and the subsequent metastasis of cancer cells (21). However, the regulation of ECM proteins is still under investigation. Whole-genome sequencing and transcriptome profiling of mouse models of breast cancer reveal the expression of these proteins and their role in metastasis (22). However, there is a dearth of information on the regulation of COL1A1 in CRC and the underlying mechanism of CRC liver metastasis.

This study aimed to investigate the molecular regulation of liver metastasis in patients with CRC. We explored the expression profile of circRNA, miRNA, and mRNA in CRC and paired adjacent normal mucosal tissues in TCGA, GEO data sets, and clinical tissues. Then, a novel upregulated circRNA, *hsa_circ_0001806*, designated circCSPP1, was identified as an oncogene and was shown to correlate with higher liver metastasis in CRC patients and to promote CRC cell liver metastasis by functioning as a ceRNA regulating COL1A1. This study demonstrated that circCSPP1 exerts an oncogenic role and may be a potential checkpoint in the diagnosis and therapy of CRC.

## Materials and Methods

### Tissues and Cell Lines

CRC tissues and corresponding adjacent non-tumorous tissues (which is 5 cm away from the tumor and confirmed by the Department of Pathology) were collected from CRC patients who underwent surgical resection without preoperative chemoradiotherapy at Xuzhou Central Hospital between October 2013 and October 2017. The human materials were obtained with the consent of the participants and approved by the ethics committee of the hospital. The human CRC cells HT29, SW480, DLD-1, LOVO, and HCT116 and the human normal mucosa cells NCM460 were purchased from the Chinese Academy of Science. All cell lines were cultured with DMEM supplemented with 10% fetal bovine serum and penicillin–streptomycin solution.

### RNA Isolation, cDNA Processing, and qRT-PCR

Total RNA was isolated from tissue and cells with TRIzol reagent (Qiagen, Hilden, Germany) according to the manufacturer's instructions. cDNA was reverse transcribed from total RNA using HiScript II Q RT SuperMix for qPCR (Vazyme, Nanjing, China). The transcripts were amplified and detected using AceQ SYBR Green PCR Master Mix (Vazyme, Nanjing, China), and the results were obtained by quantitative real-time polymerase chain reaction (qRT-PCR). The circRNA and mRNA levels were normalized to the GAPDH levels, while the miRNA levels were normalized to the U6 levels. RNase R (Epicenter Technologies, USA) was used to degrade linear mRNA. In brief, we extracted RNAs from CRC cells and divided RNA into two parts: one for RNase R digestion and another for control with digestion buffer only. The samples were incubated at 37°C for 30 min. The expression levels of circCSPP1, linearCSPP1, and GAPDH mRNA were detected by qRT-PCR. The primers used are shown in Supplementary Table 1.

### Overexpression and Silencing of Circcspp1

For upregulating and downregulating the expression of circCSPP1, full-length circCSPP1 and the short hairpin RNA (shRNA) sequence targeting circCSPP1 synthesized by GenePharma (Shanghai, China) were cloned into the lentivirus vector (Supplementary Table 1). CRC cells infected with shRNAs targeting circCSPP1 or negative control hairpin shRNA, termed shRNA 1, shRNA 2, and shControl, were verified by qRT-PCR to examine the efficiency of upregulation and downregulation of circCSPP1. The infected cells were then treated with puromycin to select the stably transfected cells. We determined that shRNA 1, which was also designated as shRNA for convenience, had the highest knockdown efficiency and used it for the next mechanistic studies.

To generate the luciferase reporter vector, the sequences of circCSPP1 and the COL1A1 3′UTR were cloned downstream of the PMIR-Reporter vector. We used a mutagenesis kit (Vazyme, Nanjing, China) to generate mutations in the miRNA-binding sites in circCSPP1 and the COL1A1 3′UTR sequence. Schematics of the above luciferase reporter vectors are provided in Supplementary Figure 1A.

### Cell Migration and Invasion Assays

In 3D migration assays, the cell suspension was harvested and pelleted by centrifugation and then plated onto Matrigel-coated plates. The Matrigel solution was prepared with DMEM supplemented with 10% FBS. After culturing at 37°C for 72 h, the cell aggregates grew in three dimensions. These results were monitored in five random fields using a microscope (100 ×).

For the cell migration Transwell assays, the cells were cultured with DMEM without FBS in the upper chamber, which was not coated with Matrigel. For the cell invasion assays, the cells were cultured with DMEM without FBS in the upper chamber coated with Matrigel. The lower chamber was filled with DMEM supplemented with 10% FBS. After incubation at 37°C for 48 h, cells located on the membrane of the lower chamber were washed with PBS, fixed with methanol, and finally stained with crystal violet solution. The numbers of cells captured in six random fields were counted by Photoshop (100 ×).

### Computational Analysis

GSE126094 is the Agilent gene chip used to examine circRNA expression changes in 10 paired CRC and adjacent mucosal tissues and was used to explore the correlation between circRNA expression dysregulation and CRC tumorigenesis and progression. The COAD and READ projects in TCGA are well-known in the study of CRC progression, and the miRNA and mRNA expression matrices were obtained from RNA-seq data from TCGA and were analyzed by edgeR. We used GSEA to obtain enriched gene sets in the KEGG data set, and 17631 mRNA expression grouped by the high or low expression of COL1A1 was used as input.

### Pull-Down Assay

Briefly, a total of 1 × 10^7^ CRC cancer cells were lysed and incubated with C-1 magnetic beads (Thermo Fisher Scientific, USA) bound to a circCSPP1 probe or oligo probe at 4°C overnight. We extracted RNA from the magnetic beads after washing the beads three times. The isolated RNA mixtures were then analyzed by qRT-PCR.

### Dual-Luciferase Reporter Assay

The wild-type (WT) or mut circCSPP1/COL1A plasmid was transfected with Lipofectamine 3000 (Sigma, USA) into CRC cells. We then co-transfected the hsa-miR-193a-5p mimic with the above plasmid into LOVO and HT29 cells. Finally, a dual-luciferase reporter system kit (Promega, USA) was used to detect the firefly and Renilla luciferase activity.

### Immunoblot Blot Assay

Immunoblot analysis was conducted as previously described (23). Briefly, total proteins were lysed by Cell lysis buffer for Western and IP (Beyotime, China) containing protease inhibitors (Beyotime, China). Total cellular protein samples were separated by 10% SDS-PAGE gel and completely transferred onto a PVDF membrane (Millipore, USA). After incubation with the primary antibodies overnight, the membranes were then incubated with goat anti-rabbit IgG H&L (Abcam, USA) for 2 h. After washing the PVDF membrane three times, we used a chemiluminescence system to detect signals and analyzed them using the FluorChem FC3 software (ProteinSimple, USA).

The primary antibodies used here were anti-rabbit COL1A1 (1:500, Abcam, catalog no. ab34710, USA), N-cadherin (1:500, Abcam, catalog no. ab202030, USA), E-cadherin (1:500, Abcam, catalog no. ab194982, USA), vimentin (1:500, Abcam, catalog no. ab92547, USA), and GAPDH (1:500, Abcam, catalog no. ab181602, USA). GAPDH was used to normalize protein loading.

### *In vivo* Metastasis Assay

A total of 120 BALB/c nude mice (6 weeks old) were randomly divided into eight groups and used for the liver metastasis model. LOVO and HT29 cells stably expressing circCSPP1 and control cells were injected into the portal vein of nude mice using a 29-G injector. Anti-NC and anti-miR-193a-5p were injected into the caudal vein of nude mice every 3 days. Mice were killed 10 weeks after inoculation or died spontaneously. All the experiments abided by the protocols put forward by the Institutional Animal Care and Use Committee of Nanjing Medical University.

### IHC Staining

Liver metastasis nodes were used for immunohistochemistry assays as described in a previous study (23). In short, deparaffinized sections were blocked for endogenous peroxidase activity and incubated with antibodies against COL1A1 according to the manufacturer's instructions. We used a digital microscope camera to obtain images of the sections (400 ×).

### Statistical Analysis

All the above experimental assays were repeated in triplicate. Data are represented as the means ± standard deviation. Student's *t*-test or two-way ANOVA was used to determine the statistical significance of the difference between two or multiple groups, and the χ^2^-test was used to assess the correlation between circCSPP1 levels and clinical features. Univariate and multivariate models were used to identify the independent risk factors for CRC patients. Data were analyzed and presented using Prism software 8.0 (GraphPad Software, USA). A *P* < 0.05 was considered to indicate a statistically significant difference, and the results are indicated as follows: ^*^P < 0.05 and ^**^P < 0.01.

## Results

### CircCSPP1 Is Upregulated in CRC

**Figure 1:**
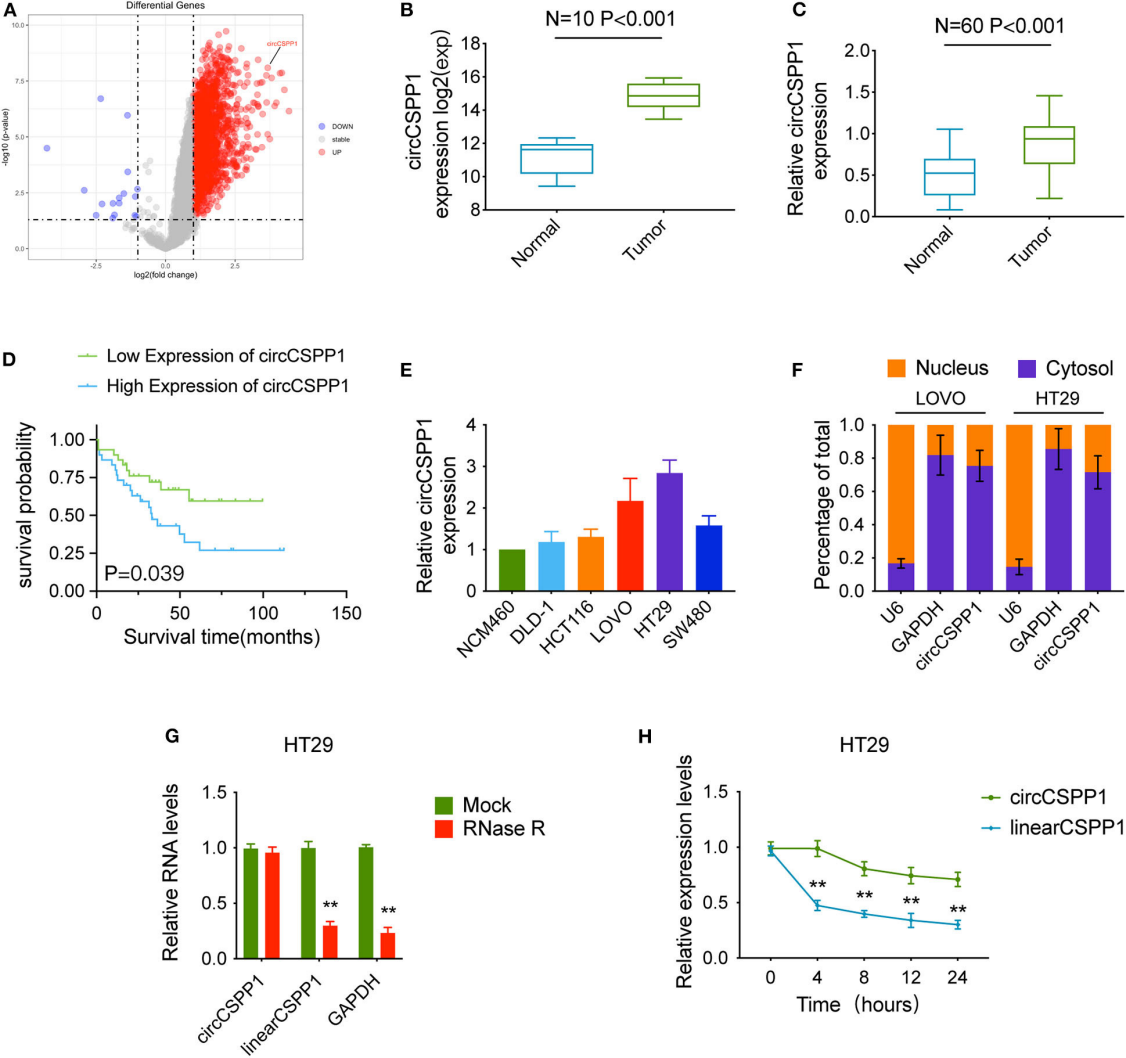
Characteristics of circCSPP1 in colorectal carcinoma. **(A)** The volcano plot visualizes the expression of circRNAs in 10 paired samples of colorectal carcinoma tissues and adjacent mucosal tissues. The red and blue dots represent dysregulated circRNAs with statistical significance. **(B)** Increased expression of circCSPP1 was shown in GSE126095 (*expression matrix is normalized and log transformed*). **(C)** The increased relative levels of circCSPP1 were confirmed in the colorectal carcinoma tissues and corresponding adjacent mucosal tissues by qRT-PCR and normalized to GAPDH levels (*N* = 60, *P* < 0.001). **(D)** Kaplan–Meier survival curve of overall survival in 60 patients with colorectal carcinoma according to circCSPP1 expression. Patients were stratified into high-expression and low-expression groups by the median expression. **(E)** The relative expression of circCSPP1 in DLD-1, HCT116, LOVO, HT29, and SW480 cells normalized to the expression of circCSPP1 in NCM460 cells. **(F)** Levels of circCSPP1 in the nuclear and cytoplasmic fractions of LOVO and HT29 cells. **(G)** Relative RNA levels of circCSPP1, linearCSPP1, and GAPDH treated with RNase R. **(H)** Relative RNA levels of circCSPP1 and linearCSPP1 in different time points. Data are presented as the means ± standard deviation (**P < 0.01).

We used the expression profiles of circRNAs (GSE126095, which contains 3,962 circRNA probes) as input to identify the differentially expressed circRNAs. A total of 1,827 dysregulated circRNAs were identified in CRC tissues, of which 1,808 circRNAs were upregulated and 19 circRNAs were downregulated (Figure 1A). Among these, a novel circRNA named circCSPP1 was differentially expressed in CRC tissues and corresponding adjacent mucosa tissues (Figure 1B). Then, the aberrant expression of circCSPP1 was confirmed using qRT-PCR (*N* = 60, *P* < 0.001; Figure 1C). Kaplan–Meier survival curves of CRC patients (*n* = 60) demonstrated that the overall survival (OS) of CRC patients with high circCSPP1 expression was significantly lower than that of low-circCSPP1-expression CRC patients (^*^P = 0.039; Figure 1D).

Next, the 60 CRC patients were grouped into circCSPP1^high^ and circCSPP1^low^ groups by the median level of circCSPP1 to investigate the clinical significance of the upregulated expression of circCSPP1 in CRC. The statistical results showed that the expression of circCSPP1 was highly correlated with the M stage but not T stage, N stage, or tumor size in CRC patients (Table 1). Further univariate and multivariate COX analyses revealed that the expression of circCSPP1 was an independent prognostic factor for CRC patients (HR = 0.09; 95% CI 0.01–0.65; *P* = 0.018; Table 2). To investigate the cellular localization of circCSPP1, we extracted and separated cytoplasmic RNA and nuclear RNA and analyzed the RNA mixture using qRT-PCR. The results revealed that circCSPP1 was preferentially located in the cytoplasm of LOVO and HT29 cells (Figure 1F). Compared with the linear form, circCSPP1 was resistant to RNase R digestion (Figure 1G), with a longer half-life (Figure 1H).

**Table 1:** Relevance analysis of circCSPP1 expression in CRC patients.

**Variable**	**All patients**	**CircCSPP1**		***P*-value**
		**Low**	**High**	
All Cases	60	30	30	
**Age (years)**				0.602
<60	26	12	14	
≥60	34	18	16	
**Gender**				0.284
Male	38	17	21	
Female	22	13	9	
**Tumor diameter (cm)**				0.606
<5	30	16	14	
≥5	30	14	16	
**TNM stage**				0.795
I+II	33	17	16	
III+IV	27	13	14	
**Depth of invasion**				0.432
T1+T2	35	19	16	
T3+T4	25	11	14	
**Lymphatic metastasis**				0.426
Yes	23	13	10	
No	37	17	20	
**Distant metastasis**				**0.010**
Yes	17	4	13	
No	43	26	17	
**CEA (ng/ml)**				0.774
<5	43	22	21	
≥5	17	8	9	
**Tumor location**				0.592
Colon	38	18	20	
Rectum	22	12	10	

*Bold values indicate P < 0.05*.

**Table 2:** Univariate and multivariate Cox regression analysis of prognostic factors for CRC patients.

**Variable**	**Univariate analysis**			**Multivariate analysis**		
	**HR**	**95% CI**	***P***	**HR**	**95% CI**	***P***
**Age (years)**						
<60	1					
≥60	0.88	0.42–1.82	0.721			
**Gender**						
Male	1					
Female	0.7	0.32–1.53	0.365			
**Tumor diameter (cm)**						
<5	1					
≥5	1.2	0.58–2.49	0.631			
**TNM stage**						
I+II	1			1		
III+IV	2.55	1.20–5.42	**0.015**	1.01	0.17–5.94	0.989
**Depth of invasion**						
T1+T2	1			1		
T3+T4	2.94	1.38–6.24	**0.005**	2.3	0.57–9.30	0.242
**Lymphatic metastasis**						
No	1			1		
Yes	2.11	1.02–4.39	**0.045**	0.62	0.16–2.51	0.506
**Distant metastasis**						
No	1			1		
Yes	3.43	1.63–7.21	**0.001**	2.95	1.10–7.91	**0.032**
**CEA (ng/ml)**						
<5	1					
≥5	1.23	0.56–2.71	0.609			
**Tumor location**						
Colon	1					
Rectum	0.66	0.29–1.50	0.324			
**Expression of circCSPP1**						
Low	1			1		
High	0.21	0.04–1.21	**0.08**	0.09	0.01–0.65	**0.018**

*Bold values indicate P < 0.05*.

### CircCSPP1 Promotes CRC Cell Migration and Invasion *in vitro*

**Figure 2:**
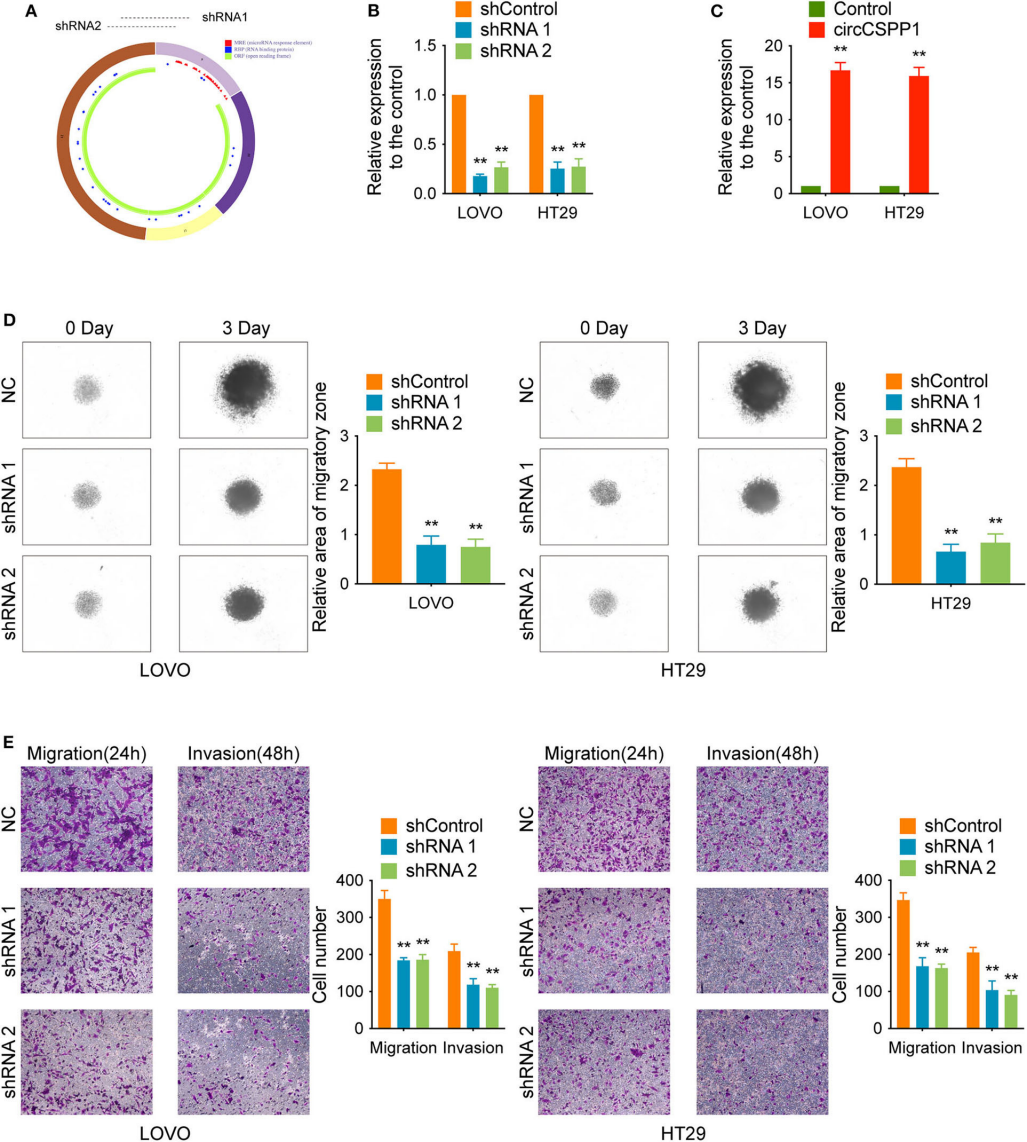
CircCSPP1 knockdown inhibited CRC cell migration and invasion *in vitro*. **(A)** CircCSPP1 is produced from the CSPP1 gene (NM_024790.6) locus containing exons 9–12. ShRNAs targeting circCSPP1 were designed according to the back-splice junction of circCSPP1. **(B,C)** Relative expression of circCSPP1 as determined by qRT-PCR in LOVO and HT29 cell lines transfected with circCSPP1 expression vector or circCSPP1-targeting shRNA. **(D)** The results of CRC cell migration were validated by 3D spheroid migration assays (six replicates per group, three independent experiments per group). **(E)** Transwell migration assay and Matrigel invasion assay of LOVO and HT29 cells after knockdown of circCSPP1. Original magnification 100 ×. Data are presented as the means ± standard deviation (**P < 0.01).

Experiments with shRNAs (shRNA 1 and shRNA 2) targeting the black-splicing region of circCSPP1 (Figure 2A) and circCSPP1 overexpression lentivirus were performed in CRC cell lines (Figure 1E) to further address the biological function of circCSPP1 in CRC. The efficiency of the lentiviral vectors was confirmed by qRT-PCR, and the stably transfected cells were used to investigate the biological function of circCSPP1 (Figures 2B,C).

**Figure 3:**
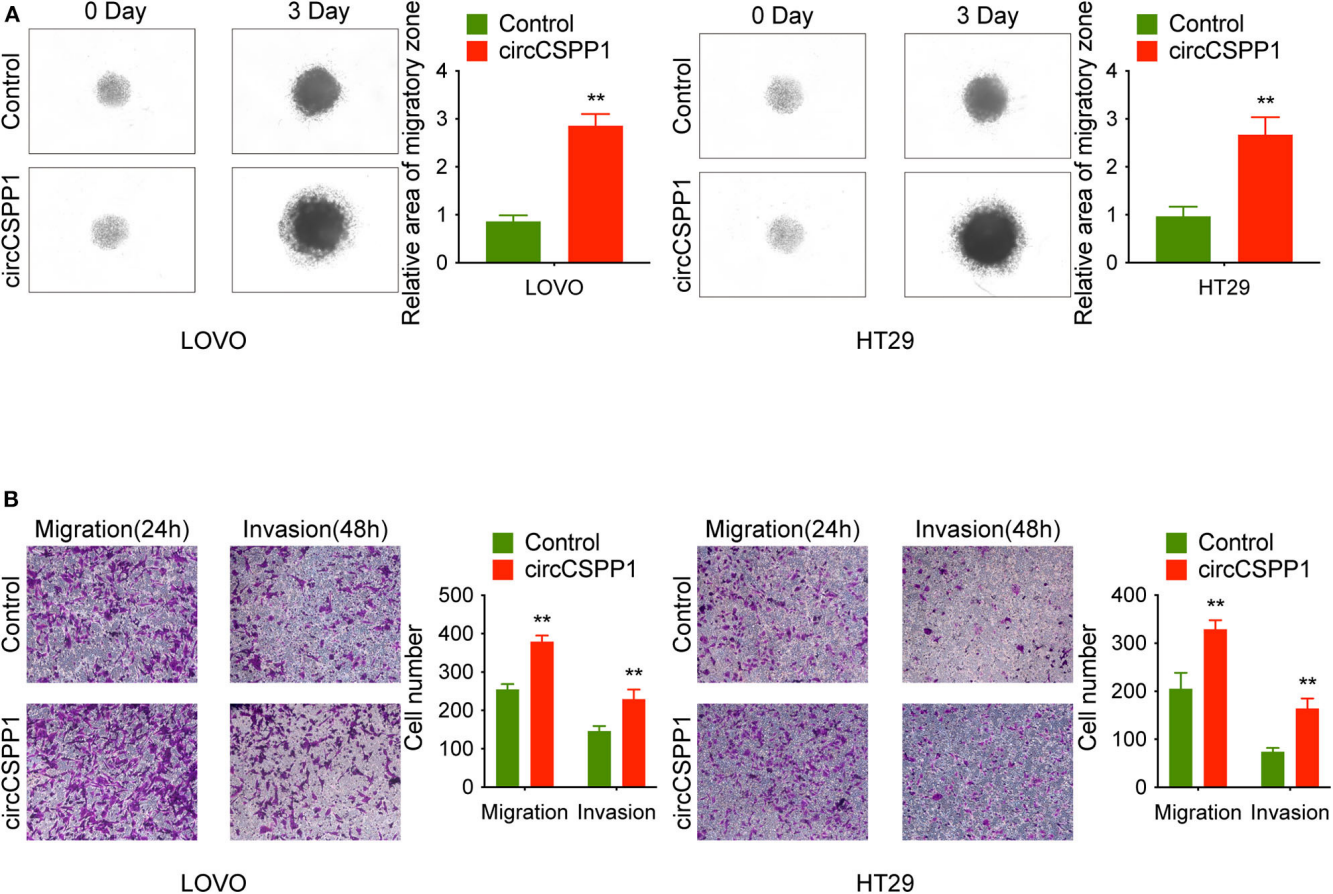
CircCSPP1 overexpression promoted CRC cell migration and invasion *in vitro*. **(A)** 3D spheroid migration assays of LOVO and HT29 cells after overexpression of circCSPP1. Images of 3D spheroid migration were taken at 0 and 24 h (six replicates per group, three independent experiments per group). **(B)** Transwell migration assay and Matrigel invasion assay of LOVO and HT29 cells after overexpression of circCSPP1. Original magnification 100 ×. Data are presented as the means ± standard deviation (**P < 0.01).

First, in the 3D migration assays, the results indicated that the upregulation of circCSPP1 significantly promoted the migration of CRC cells in Matrigel (Figure 3A). Moreover, the knockdown of circCSPP1 significantly decreased the volume of cell aggregates (Figure 2D). Next, we conducted Transwell assays to further understand the role of circCSPP1 in CRC cell migration and invasion. The results implied that circCSPP1 knockdown significantly decreased the migration and invasion of LOVO and HT29 cells compared to the control cells (Figure 2E), while the overexpression of circCSPP1 increased the migration and invasion of LOVO and HT29 cells (Figure 3B).

### CircCSPP1 Relieves the Repression of COL1A1 by miR-193a-5p

**Figure 4:**
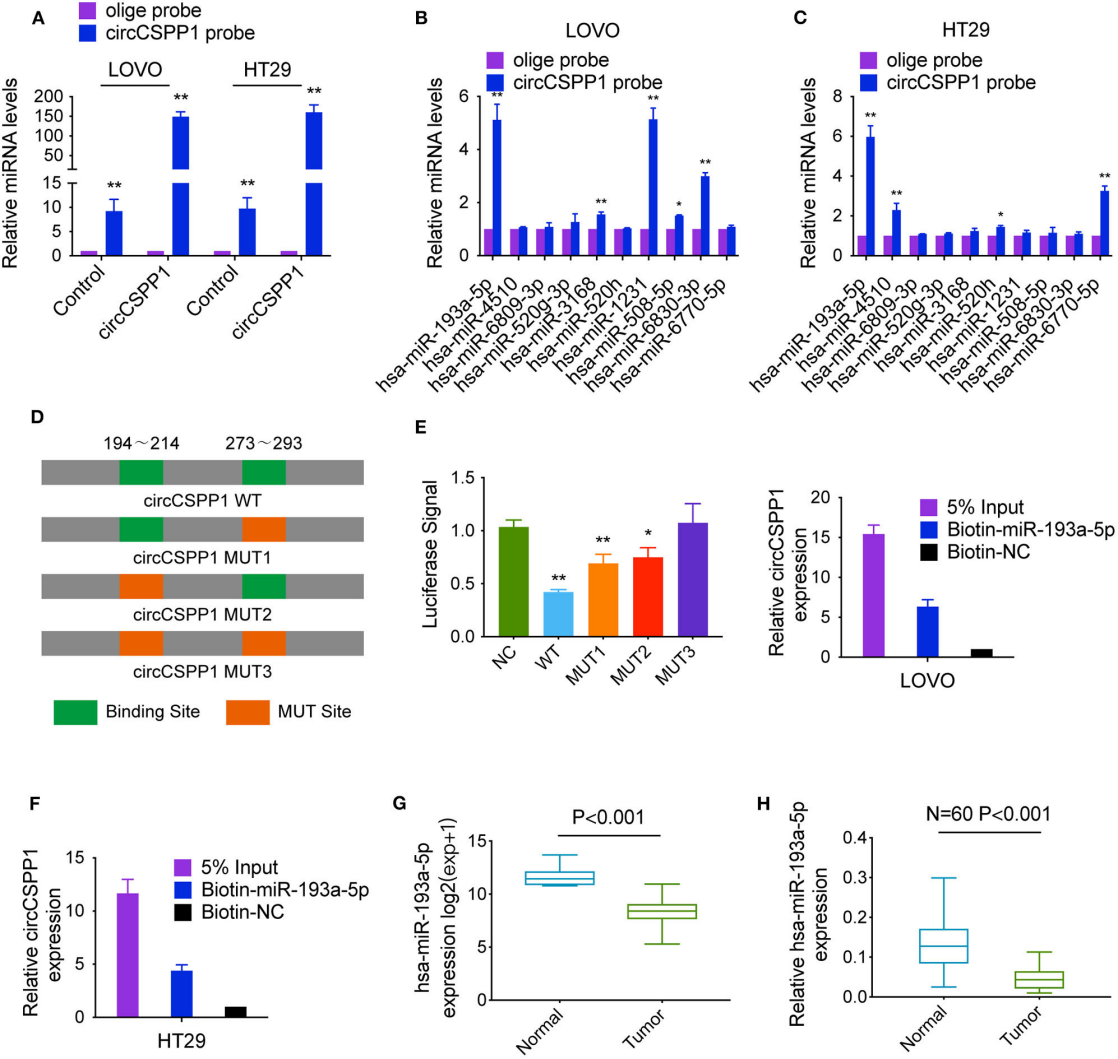
CircCSPP1 exerts its function by sponging hsa-miR-193a-5p. **(A)** Lysates prepared from LOVO and HT29 cells stably transfected with circCSPP1 or vector were subjected to RNA pull-down and tested by qRT-PCR. **(B,C)** The relative levels of 10 miRNA candidates in LOVO and HT29 lysates were detected by qRT-PCR. Multiple miRNAs were pulled down by circCSPP1, and hsa-miR-193a-5p was pulled down by circCSPP1 in both cell lines. **(D)** Dual-luciferase reporter assay was performed to determine the direct binding between circCSPP1 and hsa-miR-193a-5p based on their complementary sequences. **(E,F)** A specific biotin-labeled miR-193a-5p probe was used to capture circCSPP1 successfully relative to the NC group. **(G)** Decreased expression of hsa-miR-193a-5p was shown in TCGA (*expression matrix is normalized and log transformed*). **(H)** The increased relative levels of hsa-miR-193a-5p were confirmed in the colorectal carcinoma tissues and corresponding adjacent mucosal tissues by qRT-PCR and normalized to U6 levels (*N* = 60, *P* < 0.001). Data are presented as the means ± standard deviation (*P < 0.05 and **P < 0.01).

To identify the potential circCSPP1-sponging miRNA, we selected the top 10 candidate miRNAs according to the presence of miRNA recognition elements in the circCSPP1 sequence (Table 3). RNA pull-down assay was performed with a biotinylated circCSPP1 probe to investigate the potential circCSPP1-sponging miRNA to study the interaction between circCSPP1 and miR-193a-5p (Figure 4A). The results indicated that circCSPP1 could specifically bind to miR-193a-5p in both LOVO and HT29 cell lines but not the other miRNAs predicted by miRanda (Figures 4B,C).

**Table 3:** Top 10 targets predicted by miRanda.

**Seq1**	**Seq2**	**Tot score**	**Tot energy**	**Max score**	**Max energy**	**Strand**	**Len1**	**Len2**	**Positions**
hsa-miR-193a-5p	circCSPP1	308	−47.1	158	−24.55	824	22	432	273 194
hsa-miR-4510	circCSPP1	308	−51.93	164	−26.69	19,197	22	432	302 352
hsa-miR-6809-3p	circCSPP1	298	−32.11	156	−17.11	25,909	21	432	92 150
hsa-miR-520g-3p	circCSPP1	292	−38.3	147	−20.66	4,011	24	432	238 49
hsa-miR-3168	circCSPP1	291	−24.35	147	−14.19	15,881	17	432	169 113
hsa-miR-520h	circCSPP1	290	−29.89	146	−19.59	4,021	22	432	245 51
hsa-miR-1231	circCSPP1	286	−36.74	144	−20.86	6,962	20	432	292 1
hsa-miR-508-5p	circCSPP1	284	−46.58	144	−26.43	4,050	23	432	53 183
hsa-miR-6830-3p	circCSPP1	281	−24.93	141	−14.49	25,951	23	432	87 269
hsa-miR-6770-5p	circCSPP1	168	−24.38	168	−24.38	25,830	24	432	352

The luciferase reporter assay was used to further demonstrate the interaction between circCSPP1 and miR-193a-5p, and the results showed that miR-193a-5p significantly reduced the luciferase activity of the circCSPP1 WT reporter but not that of the mutated miR-193a-5p binding site reporters, including circCSPP1 MUT1, circCSPP1 MUT2, and circCSPP1 MUT3 (Figure 4D). In addition, we also verified the interaction of miR-193a-5p and circCSPP1 using a biotinylated miR-193a-5p probe to conversely successfully capture circCSPP1 (Figures 4E,F).

On the basis of these findings, we also found downregulated expression of miR-193a-5p in a TCGA data set, indicating that miR-193a-5p was significantly downregulated in CRC tissues and upregulated in adjacent mucosal tissues (Figure 4G). Then, the aberrant expression of miR-193a-5p was shown using qRT-PCR (*N* = 60, *P* < 0.001; Figure 4H).

**Figure 5:**
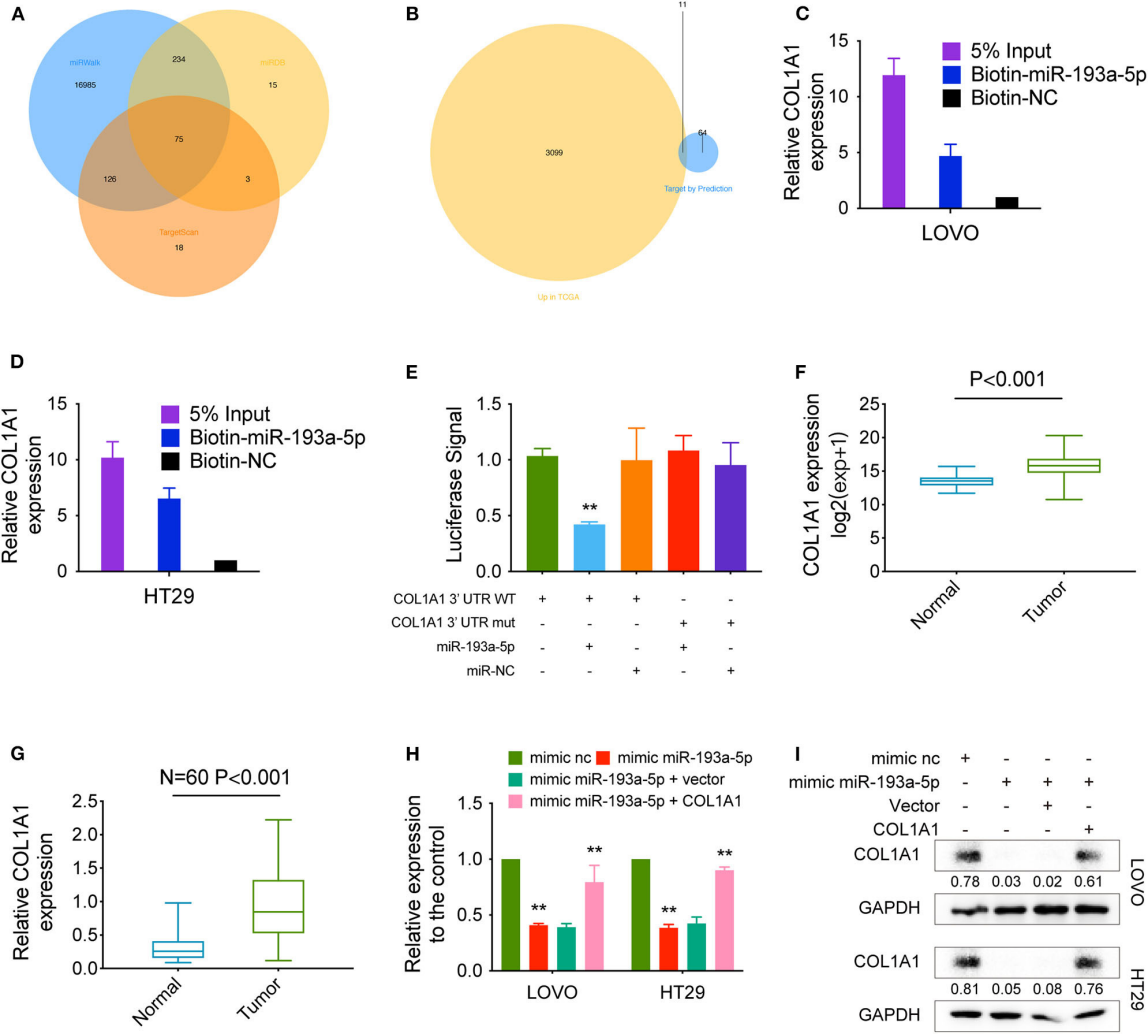
COL1A1 is a direct target of hsa-miR-193a-5p. **(A)** Venn diagram showing 75 genes that are putative hsa-miR-193a-5p targets computationally predicted by three algorithms (miRWalk, miRDB, and TargetScan). **(B)** These computed results were compared with the upregulated genes in TCGA (*P* < 0.05, logFC > 1). **(C,D)** A specific biotin-labeled miR-149-5p probe was used to capture COL1A1 successfully relative to the NC group. **(E)** A dual-luciferase reporter assay was performed to determine the direct binding between COL1A1 and hsa-miR-193a-5p based on their complementary sequences. **(F)** Increased expression of COL1A1 was observed in TCGA (*expression matrix is normalized and log transformed*). **(G)** The increased relative levels of COL1A1 were confirmed in the colorectal carcinoma tissues and corresponding adjacent tissues by qRT-PCR and normalized to GAPDH levels (*N* = 60, *P* < 0.001). **(H)** Relative mRNA levels of COL1A1 in CRC cells were determined by qRT-PCR in LOVO and HT29 cell lines transfected with the indicated plasmids. **(I)** COL1A1 protein levels in CRC cells were determined by qRT-PCR in LOVO and HT29 cell lines transfected with the indicated plasmids. Data are presented as the means ± standard deviation (**P < 0.01).

We predicted the potential targets of miR-193a-5p by three online tools (miRanda, TargetScan, and miRDB), and 75 candidate mRNAs were selected (Figure 5A). Then, the upregulated mRNAs in the TCGA data set were compared to the candidate targets, and among these, COL1A1, an EMT-pathway related gene, was chosen for further study (Figure 5B). To investigate the interaction between miR-193a-5p and COL1A1, we measured the expression of COL1A1 in the RNA mixture captured by the biotinylated miR-193a-5p probe. The positive results validated the predicted relationship (Figures 5C,D). Moreover, the luciferase reporter assay was used to further confirm the interaction between COL1A1 and miR-193a-5p, and the result showed that miR-193a-5p expression significantly reduced the luciferase activity of the COL1A1 WT reporter but not that of the COL1A1 MUT reporter (Figure 5E).

In addition, we also tested the dysregulation of COL1A1 in the TCGA COAD and READ data sets, revealing that COL1A1 was significantly upregulated in CRC tissues and downregulated in adjacent mucosal tissues (Figure 5F). Then, the aberrant expression of COL1A1 was confirmed using qRT-PCR (*N* = 60, *P* < 0.001; Figure 5G). Next, the mRNA and protein levels of COL1A1 were found to be altered in LOVO and HT29 cells with overexpression or knockdown of miR-193a-5p and could be restored by knockdown or overexpression of COL1A1 (Figures 5H,I).

### CircCSPP1 Regulates CRC Cell EMT Through COL1A1

**Figure 6:**
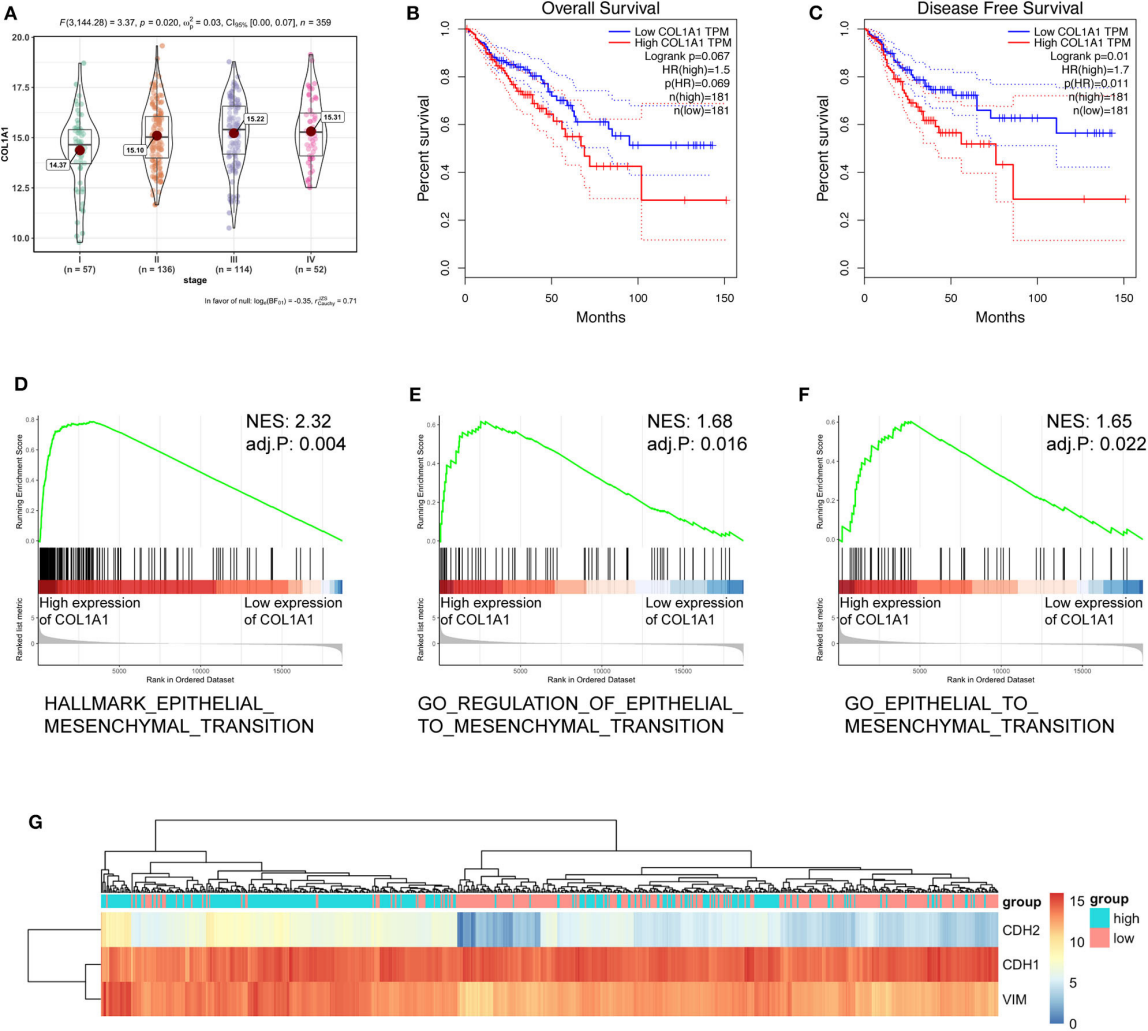
COL1A1 regulated the EMT-associated signaling pathway in TCGA. **(A)** Expression of COL1A1 in different clinical stages of colorectal carcinoma. **(B,C)** Colorectal carcinoma patients were divided into a low-COL1A1-expression group and a high-COL1A1-expression group by the median expression of COL1A1. The high COL1A1 levels correlated with poor survival rate. **(D–F)** Representative gene sets from the MSigDB in the GSEA of COL1A1 regulated genes. **(G)** A heatmap displaying the expression of EMT-associated genes categorized based on the expression of COL1A1.

To further verify the biological function of circCSPP1 in CRC, we examined the correlation between the expression of COL1A1 and clinical traits in the TCGA COAD and READ data sets. Group comparison revealed that high COL1A1 mRNA expression was significantly correlated with advanced clinical stages and increased with the progression of carcinoma (Figure 6A). Kaplan–Meier survival curves of CRC patients in TCGA demonstrated that the OS and disease-free survival (DFS) of COL1A1 high-expression CRC patients was lower than that of the COL1A1 low-expression CRC patients (Figures 6B,C). The results of GSEA showed that the EMT-related gene sets, including hallmark epithelial mesenchymal transition, GO regulation of epithelial-to-mesenchymal transition, and GO epithelial-to-mesenchymal transition, were significantly enriched (Figures 6D–F). In addition, we grouped the expressions of CDH1, CDH2, and VIM according to the expression of COL1A1. The results indicated that COL1A1 was positively co-expressed with CDH2 and VIM (Figure 6G). All of the above bioinformatics analyses indicated the EMT-regulating role of COL1A1 in CRC, which has been primarily studied by previous work (11, 12), contributed to the subsequent study of circCSPP1 in cell migration regulation.

**Figure 7:**
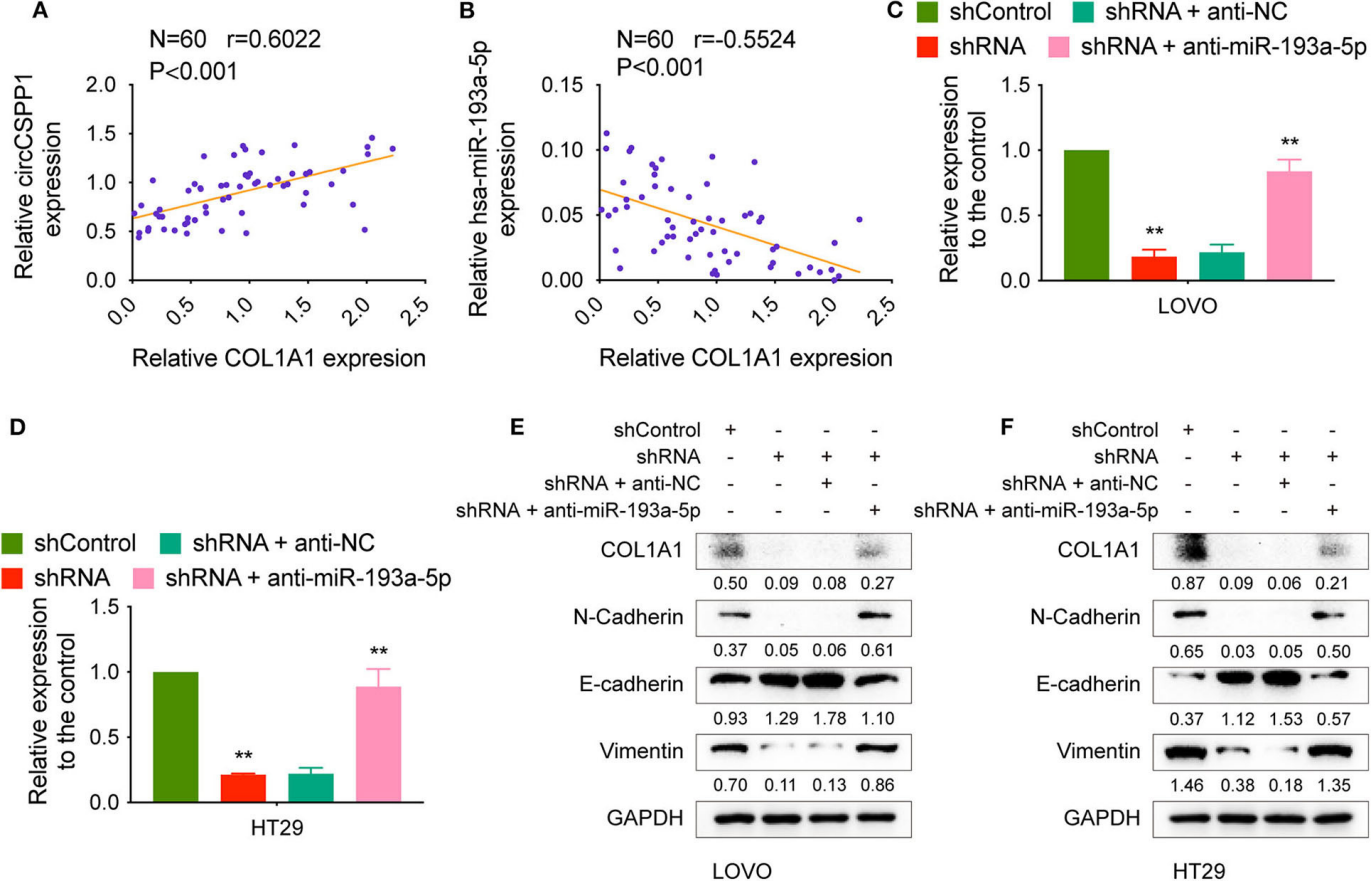
CircCSPP1 regulated the EMT-associated signaling pathway. **(A)** Pearson correlation analysis was performed to determine the correlation between the transcriptional levels of circCSPP1 and COL1A1 in colorectal carcinoma tissues. **(B)** Pearson correlation analysis was performed to determine the correlation between the transcriptional levels of hsa-miR-193a-5p and COL1A1 in colorectal carcinoma tissues. **(C,D)** Relative mRNA levels of COL1A1 in CRC cells were determined by qRT-PCR in LOVO and HT29 cell lines transfected with the indicated plasmids. **(E,F)** COL1A1 protein levels in CRC cells were determined by qRT-PCR in LOVO and HT29 cell lines transfected with the indicated plasmids. Data are presented as the means ± standard deviation (**P < 0.01).

The correlation between COL1A1 expression and the transcriptional levels of circCSPP1 and miR-193a-5p was then tested in CRC tissues, and the results showed that COL1A1 was positively correlated with circCSPP1 and negatively correlated with miR-193a-5p at the RNA level (Figures 7A,B).

We transfected LOVO and HT29 cells with shControl or shRNA targeting circCSPP1 with or without anti-NC or anti-miR-193a-5p and then examined the RNA and protein changes. The mRNA levels of COL1A1 decreased after knockdown of circCSPP1 and could be reversed by downregulation of miR-193a-5p (Figures 7C,D). The same outcome with COL1A1, N-cadherin, E-cadherin, and vimentin protein levels validated that circCSPP1 functioned as a ceRNA by targeting miR-193a-5p to regulate the expression of COL1A1 and the process of epithelial-to-mesenchymal transition (Figures 7E,F). Overexpression of COL1A1 could restore the migration and invasion abilities of LOVO and HT29 cells after being transfected with shRNA (Supplementary Figure 1B).

### CircCSPP1 Promotes Tumor Cell Liver Metastasis *in vivo*

**Figure 8:**
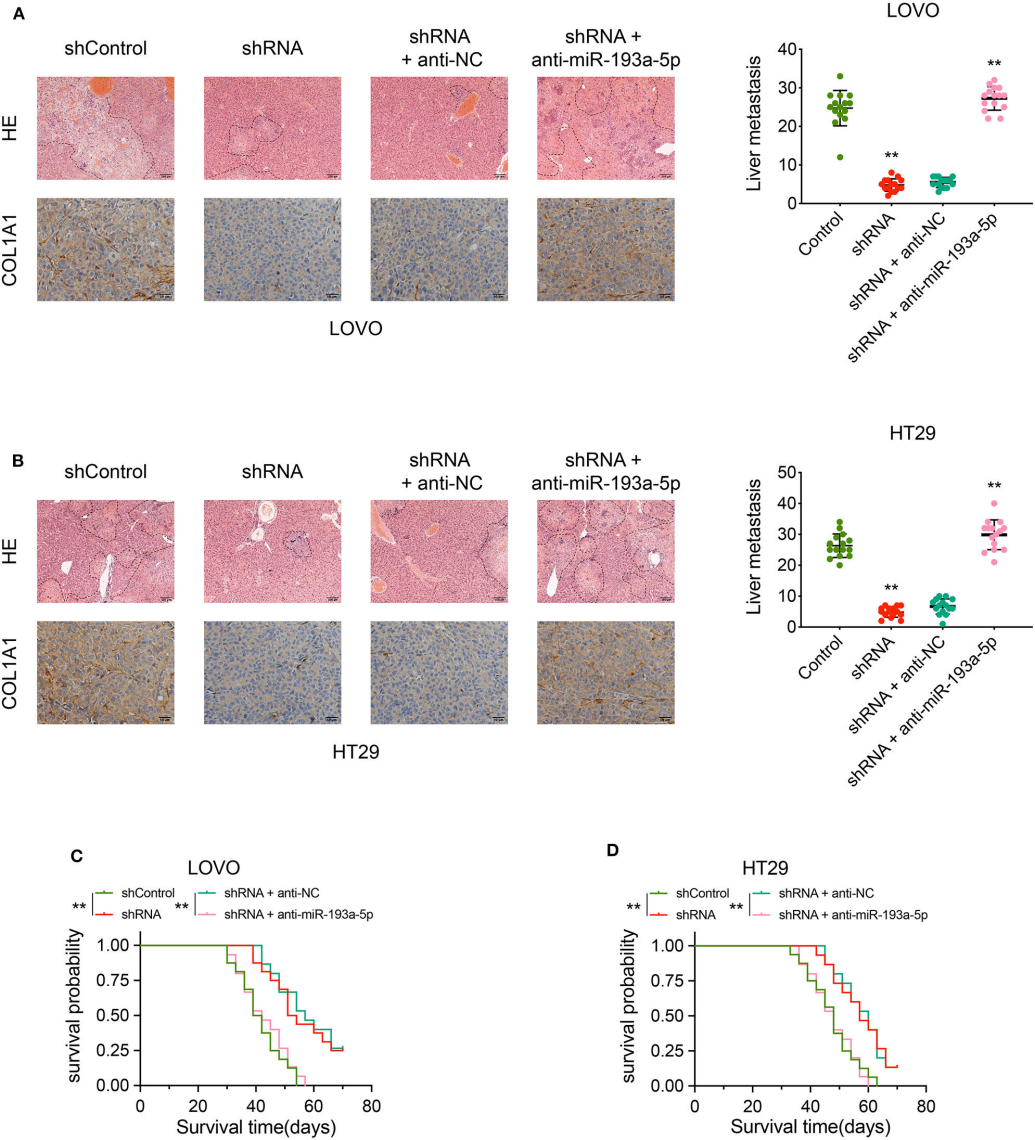
CircCSPP1 promoted CRC cell liver metastasis *in vivo* by acting as a sponge of hsa-miR-193a-5p and then upregulating the expression of COL1A1. **(A)** Left panel: representative H&E-stained images of liver metastatic nodules (upper panel, original magnification 100 ×) and IHC staining of COL1A1 in liver metastases (lower panel, original magnification 400 ×) in four treatment groups (LOVO cells). Right panel: number of liver metastatic foci was counted under a microscope. **(B)** Left panel: representative H&E-stained images of liver metastatic nodules (upper panel, original magnification 100 ×) and IHC staining of COL1A1 in liver metastases (lower panel, original magnification 400 ×) in four treatment groups (HT29 cells). Right panel: number of liver metastatic foci was counted under a microscope. **(C,D)** OS of each group of mice injected with LOVO or HT29 cells. Original magnification 200 ×. Data are presented as the means ± standard deviation (**P < 0.01).

The biological effect of circCSPP1 in COL1A1 regulation and liver metastasis was further confirmed *in vivo* using liver metastasis models with adoptive cell transfer into nude mice via the portal vein using LOVO and HT29 cells infected with the indicated vectors. The results were analyzed by H&E staining and immunohistochemical (IHC) staining, respectively. The protein levels of COL1A1 and liver metastasis regulated by circCSPP1 and miR-193a-5p were in the same pattern as observed *in vitro* (Figures 8A,B). In addition, the OS of mice was increased in the circCSPP1 knockdown groups with or without anti-NC compared to the shControl and shRNA with anti-miR-193a-5p groups (Figures 8C,D). By and large, circCSPP1 regulated COL1A1 expression to promote CRC cell metastasis.

## Discussion

The differential expression profiling of circRNAs is a prerequisite for the identification of novel tumor oncogenic factors or antitumor factors, as well as in clarifying their biological functions and underlying mechanisms (24). In this study, we identified circCSPP1 as a markedly dysregulated circRNA in CRC tissues, which was elevated with the increase in liver metastasis risk. Gain- and loss-of-function experiments revealed that circCSPP1 promoted tumor cell migration and invasion *in vivo* and *in vitro*. CircCSPP1, functioning as a ceRNA by competitively binding to miR-193a-5p, canceled out the endogenous suppressive effect of miR-193a-5p on its target mRNA COL1A1. Overexpression of COL1A1 could significantly promote the process of epithelial-to-mesenchymal transition and then drive cell migration and invasion, indicating that circCSPP1 promotes CRC cell metastasis via the ceRNA mechanism.

Genetic and protein alterations in specific signaling pathways are often observed in cancers and are thought to be associated with tumor growth and development (25). Cell EMT progression is universal in diverse cancer types (26). CircRNAs are often reported to be involved in the regulation of cell migration and invasion (27–29). To identify potential cell EMT regulators, we used bioinformatics analysis to filter upregulated and downregulated circRNAs and candidate liver metastasis targets. Among these findings, we determined that circCSPP1 was downregulated in adjacent normal colorectal mucosal tissues but upregulated in CRC tissues and could promote cell migration, invasion, and cell EMT progression *in vitro* and *in vivo*, indicating that circCSPP1 is a potential cancer liver metastasis-related gene in CRC.

COL1A1, a major component of collagen type I, is upregulated and is associated with tumor metastasis in various cancers, such as breast cancer (14), hepatocellular carcinoma (17), gastric cancer (15), CRC (12), non-small-cell lung cancer (13), prostate cancer (18), and cervical cancer (16). In this study, we found that COL1A1 was upregulated in CRC tissues, and bioinformatic analysis revealed that COL1A1 promoted cell EMT progression and was highly correlated with the OS and DFS of CRC patients. Remarkably, circCSPP1 could upregulate the mRNA levels of COL1A1, and the tumor promoter role of circCSPP1 in cell migration and invasion could be almost offset by downregulating COL1A1. Moreover, increased expression of COL1A1 in CRC cells was significantly associated with higher liver metastasis risk and lower survival rate *in vivo*, which suggests that high levels of circCSPP1 are associated with liver metastasis.

The ceRNA network theory presumes that circRNA, lncRNA, and mRNA share the same miRNA response elements (MREs), binding to miRNAs and enhancing the expression of each other (30). In this study, we used various bioinformatic software to filter target miRNAs that shared the same MREs as circCSPP1 and COL1A1. In addition, we performed biotinylated RNA/miRNA pull-down assays to examine the competitive binding activities of circCSPP1 and COL1A1 to miR-193a-5p. Further luciferase reporter assays indicated that miR-193a-5p could significantly reduce the luciferase activity of circCSPP1. All of these positive results revealed that circCSPP1 and COL1A1 could directly bind to miR-193a-5p. Rescue experiments *in vivo* and *in vitro* showed that circCSPP1 significantly suppressed the effects of miR-193a-5p on COL1A1, suggesting that circCSPP1 may regulate COL1A1 expression in CRC by functioning as a ceRNA to miR-193a-5p.

miR-193a-5p has been studied in several tumors and plays a vital role in the post-transcriptional regulation of signaling pathway proteins (31–41). For example, miR-193a-5p suppressed tumor cell metastasis and epithelial-to-mesenchymal transition by targeting WT1-E-cadherin in non-small-cell lung cancers (41). However, the roles of miR-193a-5p in CRC cell liver metastasis remain under investigation. In this study, we found that miR-193a-5p was sponged by circCSPP1, resulting in less miR-193a-5p targeting COL1A1 and enhancing CRC cell migration and invasion. The existence of the circCSPP1/miR-193a-5p/COL1A1 axis improves our understanding of the underlying mechanism of CRC cell liver metastasis.

Finally, we investigated the downstream signaling pathway of COL1A1 that contributes to circCSPP1-mediated biological function. Knockdown of circCSPP1 decreased the expression of N-cadherin and vimentin and increased the expression of E-cadherin by downregulating COL1A1. N-Cadherin, a mesenchymal cadherin, is strongly correlated with metastatic dissemination (42). E-Cadherin, a single transmembrane protein, mediates cell–cell adhesion in a strictly Ca^2+^-dependent manner (43). Vimentin, a mesenchymal marker, enables tumor cells to delaminate from the primary tumor and invade locally (44). These proteins are involved in epithelial-to-mesenchymal transition. It has been reported that N-cadherin, E-cadherin, and vimentin are dysregulated in CRC tissues and associated with poorer OS (45). However, the potential regulation of EMT in CRC is still under exploration. In our study, the results partially reveal that EMT progression can be regulated by circCSPP1 in CRC.

Conclusively, our study determined that circCSPP1, competitively binding to miR-193a-5p, offsets the inhibitory effect of miR-193a-5p on COL1A1 and then promotes CRC cell migration, invasion, and metastasis. These findings provide a novel insight into exploring the molecular mechanism of development and progression of CRC and a potential therapeutic target for CRC.

## Data Availability Statement

Publicly available datasets were analyzed in this study, these can be found in the Cancer Genome Atlas (https://portal.gdc.cancer.gov/); the NCBI Gene Expression Omnibus (GSE126094).

## Ethics Statement

The studies involving human participants were reviewed and approved by the Ethics Committee of Xuzhou Central Hospital. The patients/participants provided their written informed consent to participate in this study. This animal study was reviewed and approved by the Institutional Animal Care and Use Committee of Nanjing Medical University.

## Author Contributions

QW: manuscript and main experiments. LS: figures and data analysis. YS, XW, and JY: design of this study. KS, BY, GC, CK, JF, ZM, XL, XuZ, YF, XJ and XiZ: assist of experiment and revise of manuscript. All authors contributed to the article and approved the submitted version.

## Conflict of Interest

The authors declare that the research was conducted in the absence of any commercial or financial relationships that could be construed as a potential conflict of interest.

## Supplementary Material

Supplementary Figure 1 **(A)** Schematic of CSPP1 3′UTR wild-type (WT) and mutant (MUT1&2) luciferase reporter vectors, COL1A1 3′UTR wild-type (WT) mutant (MUT) luciferase reporter vectors are shown. **(B)** Transwell migration assay and Matrigel invasion assay of LOVO and HT29 cells infected with shRNA after overexpression of COL1A1 or not. Original magnification 100 ×. Data are presented as the means ± standard deviation.

Supplementary Table 1 Detailed information of oligonucleotide sequences.
