# Supplementary figures and images for: CircCSPP1 Functions as a ceRNA to Promote Colorectal Carcinoma Cell EMT and Liver Metastasis by Upregulating COL1A1
Source: Front Oncol. 2020 Jun 16;10:850. doi: 10.3389/fonc.2020.00850 (PMC7308451; doi:10.3389/fonc.2020.00850)

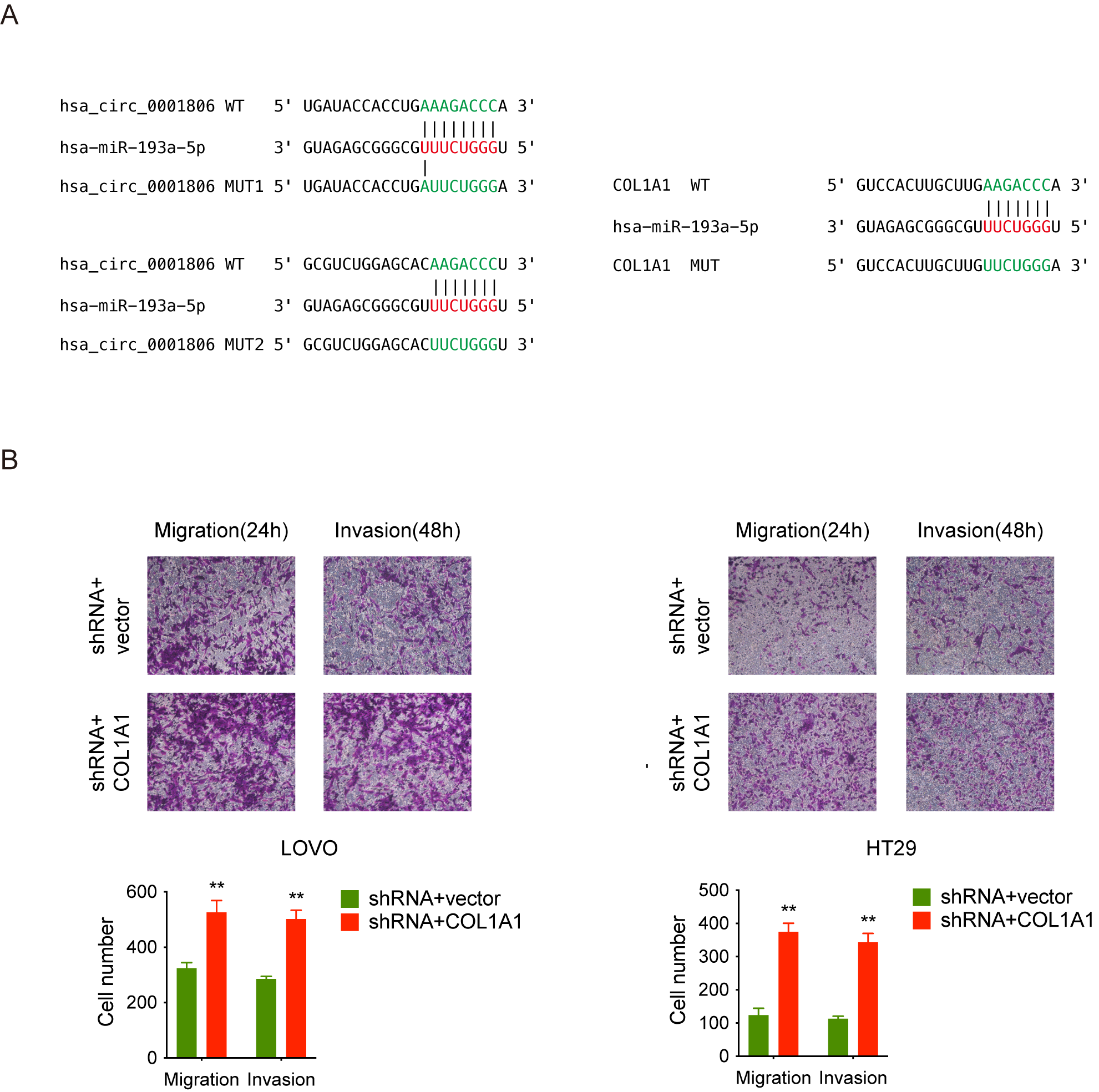

Supplement: Supplementary Figure 1 — (A) Schematic of CSPP1 3′UTR wild-type (WT) and mutant (MUT1&2) luciferase reporter vectors, COL1A1 3′UTR wild-type (WT) mutant (MUT) luciferase reporter vectors are shown. (B) Transwell migration assay and Matrigel invasion assay of LOVO and HT29 cells infected with shRNA after overexpression of COL1A1 or not. Original magnification 100 ×. Data are presented as the means ± standard deviation. [file Image_1.TIF]
