# Supplementary material for: CircCSPP1 Functions as a ceRNA to Promote Colorectal Carcinoma Cell EMT and Liver Metastasis by Upregulating COL1A1
Source: Front Oncol. 2020 Jun 16;10:850. doi: 10.3389/fonc.2020.00850 (PMC7308451; doi:10.3389/fonc.2020.00850)
Supplement: Supplementary Table 1 — Detailed information of oligonucleotide sequences. [file Table_1.DOCX]

| **Supplementary Table 1 Detailed information of oligonucleotide Sequences** | | |
| --- | --- | --- |
| **Gene** | **Sequence** |  |
| circCSPP1 | Forward | 5'-CCATCCCATCAGTTCATCCT-3' |
|  | Reverse | 5'-CCCTGCAAAAGGACTACAGG-3' |
| COL1A1 | Forward | 5'-TGACCTCAAGATGTGCCACT-3' |
|  | Reverse | 5'-CCTCTTGTCCTTGGGGTTCT-3' |
| GAPDH | Forward | 5'-ACAACTTTGGTATCGTGGAAGG-3' |
|  | Reverse | 5'-GCCATCACGCCACAGTTTC-3' |
| U6 | Forward | 5'-GCTTCGGCAGCACATATACAAAAT-3' |
|  | Reverse | 5'-CGCTTCACGAATTTGCGTGTCAT-3' |
| hsa-miR-193a-5p | Forward | 5'-TGCGGGCGAGATGAGTC-3' |
|  | Reverse | 5'-CAGTGCGTGTCGTGGAGT-3' |
| hsa-miR-4510 | Forward | 5'-TGAGGGAGTAGGATGTATGGT-3' |
|  | Reverse | 5'-CAGTGCGTGTCGTGGAGT-3' |
| hsa-miR-6809-3p | Forward | 5'-CTTCCCAGGTCGTATCCAGT-3' |
|  | Reverse | 5'-CAGTGCGTGTCGTGGAGT-3' |
| hsa-miR-520g-3p | Forward | 5'-AGTGCTTCCCTTTAGAGTGTG-3' |
|  | Reverse | 5'-CAGTGCGTGTCGTGGAGT-3' |
| hsa-miR-3168 | Forward | 5'-AGTTCTACAGTCAGACGTCGT-3' |
|  | Reverse | 5'-CAGTGCGTGTCGTGGAGT-3' |
| hsa-miR-520h | Forward | 5'-TGCTTCCCTTTAGAGTGTCGT-3' |
|  | Reverse | 5'-CAGTGCGTGTCGTGGAGT-3' |
| hsa-miR-1231 | Forward | 5'-ACAGCTGCGTCGTATCCA-3' |
|  | Reverse | 5'-CAGTGCGTGTCGTGGAGT-3' |
| hsa-miR-508-5p | Forward | 5'-ACTCCAGAGGGCGTCAC-3' |
|  | Reverse | 5'-CAGTGCGTGTCGTGGAGT-3' |
| hsa-miR-6830-3p | Forward | 5'-CCTTGCAGGTCGTATCCAG-3' |
|  | Reverse | 5'-CAGTGCGTGTCGTGGAGT-3' |
| hsa-miR-6770-5p | Forward | 5'-GTGAGTCGTATCCAGTGCAA-3' |
|  | Reverse | 5'-CAGTGCGTGTCGTGGAGT-3' |
| mimics miR-193a-5p |  | 5'-UGGGUCUUUGCGGGCGAGAUGA-3' |
| mimics nc |  | 5'-UUCUCCGAACGUGUCACGUTT-3' |
| anti-miR-193a-5p |  | 5'-UCAUCUCGCCCGCAAAGACCCA-3' |
| anti-nc |  | 5'-UUCUCCGAACGUGUCACGUTT-3' |
| circCSPP1 shRNA 1 | sense | 5’-GATCCGCAGAGGGUCACGAGGUCUGUUTTCAAGA GAAACAGACCUCGUGACCCUCUGCTTTTTTG-3' |
|  | antisense | 5’-AATTCAAAAAAGCAGAGGGUCACGAGGUCUGUUT CTCTTGAAAACAGACCUCGUGACCCUCUGCG-3' |
| circCSPP1 shRNA 2 | sense | 5’-GATCCGUACCACAGAGGGUCACGAGGUTTTTCAA GAGAACCUCGUGACCCUCUGUGGUATTCTTTTTTG-3' |
|  | antisense | 5’-AATTCAAAAAAGUACCACAGAGGGUCACGAGGUTT TCTCTTGAA ACCUCGUGACCCUCUGUGGUATTCG-3' |
| Control shRNA | sense | 5'-GATCCGTTCTCCGAACGTGTCACGTTTCAAGAGAA CGTGACACGTTCGGAGAACTTTTTTG-3' |
|  | antisense | 5'-AATTCAAAAAAGTTCTCCGAACGTGTCACGTTCTCT TGAAACGTGACACGTTCGGAGAACG-3' |
| circCSPP1 siRNA 1 | sense | 5'-CAGAGGGUCACGAGGUCUGUUTT-3' |
|  | antisense | 5'-AACAGACCUCGUGACCCUCUGTT-3' |
| circCSPP1 siRNA 2 | sense | 5’-UACCACAGAGGGUCACGAGGUTT-3' |
|  | antisense | 5'-ACCUCGUGACCCUCUGUGGUATT-3' |
| Control siRNA | sense | 5'-UUCUCCGAACGUGUCACGUTT-3' |
|  | antisense | 5'-ACGUGACACGUUCGGAGAATT-3' |
